# Gynæcological and Obstetrical Society of Baltimore

**Published:** 1889-02

**Authors:** 


*Regular Meeting, November 13, 1888.*

The President, Dr. Opie, in the Chair.

Dr. Thomas Opie reported

### TWO OVARIOTOMIES WITH UNUSUAL COMPLICATIONS.

Case 1.—Mrs. B., married at 25 years of age. Nine months afterwards, in March, 1883, I was called to see her when suffering from an acute retroflexion of the uterus at the middle of the third month of gestation. Manual efforts to replace the organ failed. A colpeurynter was introduced at 8 p.m. and on my visit the following morning, the uterus was replaced. At the expiration of seven months of pregnancy, she had a premature delivery. The child died in 24 hours. Conception occurred again in a year and she aborted at the end of the second month. Upon the recurrence of menstruation, she began to have trouble with the right ovary. At every ovulating period this ovary became swollen to the size apparently of a hen's egg. Later it suddenly disappeared and was followed by tenderness, nausea and vomiting. Her pulse was about 120 and temperature ranged from 102° to 103°. These symptoms, lasting for three or four days, were repeated at four or five successive ovulations. The case drifted away from me and returned in March, 1888. She said she first noticed an enlargement in the right side in October, 1885, which gradually grew until it filled the abdomen.

Ovariotomy was performed April 29th, 1888. When the peritoneum was opened, it was found to contain three gallons of ascitic fluid, a sample of which was taken for examination.

The patient took chloroform so badly, that ether was substituted. On the escape of the fluid, the respiration, which was so embarrassed that it threatened to debar the progress of the operation, grew fuller and slower, and the anæsthesia became entirely satisfactory. The sac was emptied and a sample of its contents secured. The solid part of the tumor was large and the adhesions to the intestines very extensive. A segment of the sac—about six inches square—which could not be separated from the intestines without great risk, was left. The action on the part of the right ovary, prior to the development of the ovarian tumor, was peculiar. Either there was an abnormal maturation or rupture of an ovisac or a thin wall cyst acting as the dermoid cyst sometimes does, ruptured and induced a peritonitis monthly, at the time of ovarian activity. This was likely the beginning of the ascites and the Drysdale corpuscles in the peritoneal fluid may have been derived from this source.

I subjoin the microscopic examination of the contents of the peritoneal and sac cavities, as made by Dr. N. G. Keirle.

*Pathological histology of the ovarian tumor removed by Dr. Opie.*—This ovarian growth is an ovarian cystoma described by Rindfleisch ("Pathological Histology," vol. ii., p. 180. Syd. Soc. Ed,) as Form II. Single ovary usually affected, a few large cysts and numerous smaller ones; "the surface of the tumor is nearly always connected with the peritoneum by a large number of inflammatory adhesions traversed by veins of considerable size;" cyst walls thin and lacerable and contents mixed with blood and blood pigment; microscopically these cysts are lined with epithelium and traversed with bands of connective tissue and throughout the structure are numerous corpuscles described by Drysdale, which, perhaps, are the granular cells, normal in the inter-follicular stroma, as described by His in Stricker's Histology, vol. ii., p. 195, Syd. Soc. Ed.: This tumor is a cystoma due to colloid degeneration of the ovarian stroma; the cyst contents coagulate firmly, on addition of nitric acid, due, perhaps, to admixture of blood serum.

Ascitic fluid from same case contains numerous Drysdale corpuscles which may indicate that the abdominal dropsy supervened upon—was caused by escape of irritant cyst contents: fluid firmly coagulates with nitric acid.

Case II.—Mrs. C. was operated upon Nov. 4th, 1888, for the removal of an advanced cystomata. In July last she fell down a steep stairway and struck her abdomen against a chair. Acute peritonitis ensued from which she was critically ill. A considerable amount of solid elements was recognizable in the tumor in the right hypochondrium at the point where the blow was received.

The incision in this, as in the first case, was extended above the umbilicus and was seven inches long. It was made directly through the umbilicus, its length having been essential to the separation of the adhesions and the removal of the solid part of the tumor. Three-fourths of the sac wall was adherent to the structures around it. A section of it about six inches long and four wide, which could not be detached from the intestines, was trimmed closely and left *in situ*. Having staunched all hæmorrhage and cleansed the abdominal cavity, the whole surface of the peritoneum and the remaining part of the sac were insufflated with iodoform, selected with a view to its purity, dryness and thoroughness of levigation.

The peritoneum was evenly closed with fine Chinese silk by a continuous suture and the walls of the wound so secured by interrupted silver wire sutures a half inch apart, as to fit together in the successive layers, structures of the same kind.

In this, as in the other case, Keith's glass drainage tube was inserted, in view of the remaining segment of the cyst wall and the extensive peritoneal traumatism. The temperature rose to its highest, 102°, on the second day and the pulse ran up to 120. Vomiting and straining, abdominal tenderness and tympanites, pointed to peritonitis, which lasted for four days. The pulse continued above 100 for a week and was the last symptom of the group to abate. The incision healed in both cases by first intention. There was no discharge, except a few drops of odorless, sanious serum to exude from the drain tubes. Both tubes were removed on the 10th day and iodoform blown on the margins of the opening. I have great confidence in the restraining influence of iodoform on the exudation of serum from the wounded peritoneal surfaces. It constringes the capillaries and dries thoroughly the whole serous investment of the abdomen, in most cases doing away with the necessity for drain tubes and foreclosing the fresh wounds from receiving germs of empoisonment. In these two operations the drainage tubes might well have been omitted. Greig Smith says, when in doubt, drain. Fewer cases have died from drainage than from the want of it. It seems likely, however, that drainage tubes will be less and less used, and will belong after a while, like the clamp, only to the history of ovariotomy.

Dr. T. A. Ashby then read a paper on

LAPAROTOMY FOR ASCITES.

The object of the paper was to demonstrate the value of an exploratory laparotomy as an aid to the diagnosis and treatment of ascites in those cases where the cause of this condition could not be referred to structural changes in well-known organs as the liver, kidneys, heart or spleen, or to inflammatory conditions, but was evidently dependent upon mechanical influences. Ascites could be referred to a large number of causative influences and its successful treatment depends largely upon an ability to ascertain and remove the cause. Its dependence upon intra-abdominal and intra-pelvic growths was well recognized and its disappearance after the removal of such growths was an established fact. While such growths often demanded removal for other symptoms than those referable to the ascites, this latter condition was a plain indication in certain cases. Where the diagnosis of the cause of ascites could not be clearly established and the symptoms did not yield to the influence of drugs, exploratory laparotomy was advised as the most rational way of ascertaining the cause, and it afforded a remarkable hope of being able to remove the influence at work in the production of the effusion. Exploratory laparotomy, aseptically performed, was a safe procedure and should be undertaken when the indications clearly pointed to a mechanical cause of the ascites.

In confirmation of the value of laparotomy for this condition a case was related with the following brief history: A young lady, single, aged nineteen, began to suffer with menstrual disturbances in January, 1888. Her health became depreciated and about May 1st an abdominal enlargement was first noticed. On June 1st ascites was so pronounced and she became so distressed that paracentesis became necessary. Drugs previously administered had failed to reduce the effusion. Two gallons and a half of ascitic fluid were removed. The effusion immediately returned and by June 10th the abdomen was as large as prior to the operations. An exploratory laparotomy was now made and a wild tumor the size of a hen's egg was found in the pelvis behind the uterus. This was enucleated and removed. The effusion was referred to this cause: the tumor acting mechanically upon an important blood-vessel had occasioned the ascites. Recovery followed the laparotomy and up to the present date there has been no return of the ascites, though over five gallons had accumulated within thirty days prior to the removal of the tumor. The diagnosis of the tumor could not be made prior to the laparotomy. This discovery and removal of the cause by intra-abdominal exploration led to a recovery of the patient from the ascites. Drugs could have been of no curative value in this case, and paracentesis was only temporary and palliative in its aim. The procedure was advocated as rational in its intent and possibly curative in its results in each condition of ascites of pronounced mechanical origin.

Dr. Opie thought that Dr. Ashby was to be congratulated on the success of his laparotomy. There is, however, a great danger in laparotomies that are merely exploratory. All such cases do not turn out so happily as this one. There is a fascination in the novelty of such procedures and a danger of being swerved by them from our conservatism in surgery. Had the tumor been larger or situated elsewhere than it was, either in the abdominal or pelvic cavities, it would likely have been found after tapping either by palpation or this mode of exploration combined with vaginal or rectal touch. The recent advance in the knowledge of palpation has added to the value of the trocar. Its use as an exploratory method should not be too much depreciated, since it is simple and much safer than laparotomy, and has its legitimate sphere in the management of cystomas. It occurs sometimes that the patient has some acute disease, and tapping might be resorted to, where the more heroic operations of laparotomy or ovariotomy would be contraindicated. Again, a patient with ascites or cystomata may be too anæmic or debilitated for operation, and we tap to obtain time for tonic treatment. A singular case came under his observation a few days since, which has some bearing upon the cases recently published of ovariotomy during pregnancy. It was a lady 34 years of age who had borne 5 children. After the birth of the first child a cystoma developed and was tapped; subsequently she bore to full term two children and was tapped the second time while pregnant with her third child. The third time she was tapped was during her fifth pregnancy. Since the last birth two years have elapsed aud the abdomen is again as large as at the eighth month of pregnancy. The four children born since the first appearance of the cystoma were fully developed and vigorous at birth, and three of them are still alive.

Dr. Ashby regretted having to call in question the opinion of Greig Smith, expressed by Dr. Opie, in regard to the value of hot water as a hæmostatic. It is well-known that hot water stimulates a temporary flow of blood to the capillaries and primarily increases hæmorrhage, but this effect is only transient and is now followed by a shrinkage of the vessels and a cessation of hæmorrhage. It therefore becomes a most reliable hæmostatic agent and perhaps the best at the surgeon's command in cases of capillary bleeding. In abdominal surgery, hot water is equally useful in relieving the depression following prolonged exposure of the viscera and as a cleansing agent. It is in the speaker's opinion the best hæmostatic he had ever employed in abdominal work where there was capillary bleeding.

He admitted the force of Dr. Opie's remarks in reference to the operation of paracentesis, but in the case related by him he believed the value of an exploratory incision was fully established. The opening of the abdomen was a fairly harmless procedure and where the diagnosis was in doubt and a mechanical cause was manifest, an exploration should be made to determine, if possible, the nature of the cause, and attempt at its removal should be made if possible. He did not question the value of drugs or of the trocar in properly selected cases. Medicine should yield to surgery when its mission had proven useless and where surgical intervention gave a hope of satisfactory results.

Dr. L. E. Neale referred to one case of his own that illustrated the good result of laparotomy for ascites in a manner even more striking than the case of Dr. Ashby. It was in a colored girl who presented a uterine fibroid accompanied by ascites due to no other discoverable cause than the local irritation of the peritoneum by the tumor. Dr. Neale removed three gallons of ascitic fluid by laparotomy and at the same time took out the tubes and ovaries but left the tumor *in situ*. This was over two years ago: there has been no return of the ascites and the tumor has now entirely disappeared.

He was unable to explain satisfactorily this alteration in function of a serous membrane by substituting one irritant for another, especially where, as in his case, the primary irritant remained for a very considerable period. Laparotomy for ascites in certain cases was a well-known plan of treatment that had been thoroughly demonstrated as justifiable by the brilliant results of Mr. Lawson Tait and other laparotomists. In the present light of pathology on this subject he was at a loss to discriminate between therapy, trocar and knife, and thought each case must be decided on its own merits.

With reference to the removal of the second ovary when one had to be taken out on account of serious disease, he thought we were still in the dark. For although Mr. Tait held it to be better to remove both where any doubt existed, he had in his own experience one case to prove the exception. This was his first case of ovariotomy where a large unilateral multilocular cystoma was removed, and where the second ovary although somewhat enlarged and presenting numerous cysts about the size of a pea on its surface was, after much opposing advice by experienced laparotomists, left in with the result that the woman has since borne three fine children.

Dr. Neale exhibited specimens from his own cases where relief from hysteria-hystero-epilepsy, dysmemorrhœa, etc., had followed the removal of these so-called cystic ovaries which, upon examination by an expert pathologist, were declared to be not sufficiently diseased to account for these disorders. From his own experience he was forced to believe that the severest functional disorders might arise from ovaries that were neither macroscopically nor microscopically diseased, and he was unable always to tell what constituted a diseased ovary. This case was especially interesting, as it had been previously treated by another physician with a useless therapy and the trocar.

Dr. William Pawson Chunn read a paper on

### CHRONIC INFLAMMATION OF THE VULVO-VAGINAL GLAND.

Chronic inflammation of the vulvo-vaginal gland is a rare disease, and not very frequently observed. It may be that while listening to these remarks others present may recall similar recollections. Such recollections are what is desired and which give to a discussion its value and make it of interest. Cases sometimes occur where acute attacks of inflammation do not subside in the usual way, but continue with frequent exacerbations, leaving the gland in a permanently pathological condition. For illustration, we may take the tonsil, which inflames, swells, suppurates and bursts, and which continues so to do until finally extirpated. My attention was first called to this trouble by a patient who complained of a mucous discharge about the vulva. She had had gonorrhœa sometime before and considered herself well, with the exception of the symptom mentioned. The discharge appeared at irregular intervals and was of a viscid, glairy nature. Naturally thinking it was from the uterus or vagina, I made a vaginal examination, but failed to detect the cause; the gland, at the time, being hid behind the speculum. A subsequent examination, however, was successful and a hard, nodular swelling was located at the site of the right vulvo-vaginal gland. The swelling was perforated by two or three small openings, which, upon pressure, gave vent to the peculiar fluid already mentioned. It was a source of considerable trouble to the patient and beside producing an eczematous eruption, was the cause of dyspermasia. This condition was treated by applications of iodine, introduced into the sinuses, and, although some improvement resulted, a cure was not established. A second case, somewhat similar, came under my notice about the same time and with the same symptoms, but without any discharge. This proved to be a cyst of the gland, and was afterwards extirpated, much to the relief of the patient. A cyst, however, is not to be considered under the head of chronic inflammation, and is only mentioned as an aid to a proper diagnosis. A short time ago I was called in consultation to see a lady who was supposed to be suffering with a lupus or ulcer on the vulva, accompanied by a continual discharge, and pain on locomotion. After arriving at the house I made an examination, and found on the left side of the vulva, low down, and slightly within, the hard fibrous mass already spoken of. There were numerous small openings giving out a discharge upon pressure. The mass was hard and about the size of a walnut. I advised extirpation and, upon a subsequent occasion, operated in the following way: A vesical incision was made over the most prominent part of the swelling, through the skin, which was then dissected aside and, a pair of vulsellum forceps being used to hold the mass, the whole gland was rapidly removed with scissors. There was some hæmorrhage and one artery had to be tied. The upper part of the incision was then closed with three silk sutures, the lower angle being left open for drainage. The operation proved successful, and so far as I know the patient still continues well.

Dr. B. B. Browne had met with a case (*Maryland Medical Journal*, vol. viii., p. 399.) in which the gland had ulcerated into the rectum. The patient had had an inflammation of the left labium: an abscess formed which broke on the mucous surface of the vulva and discharged a large amount of pus. The suppuration continued for about two months when she noticed that flatus escaped through the fistula and shortly afterward fæcal matter also made its appearance. At this time she consulted me and I found, upon examination, a fistulous opening leading from the labium majus to the lower bowel, passing in the direction of Bartholin's gland, thus forming a *recto-vulvar fistula.* In order to avoid, as far as possible, any weakening of the perineum, the incision was made directly outwards from the mucous surface and to the left of the median line. The fistula healed up from the bottom, and the integrity of the perineum was not in the least impaired.

Dr. Thomas Opie had met with two cases of painful over-distention of the vulvo-vaginal gland, which he thought might have eventuated in abscess, if they had not been relieved. He thought that such abscesses were usually produced by over-distention of the gland, the duct having been obstructed by catarrhal or specific vulvitis. By seizing the gland between the thumb and forefinger and making firm pressure in the direction of the duct, the superficial obstruction at the exit of the duct was overcome. Having once started the flow it soon becomes easy to relieve entirely the distended gland. The relief in both cases was permanent.

Dr. Chunn explained that his cases were much more chronic than those related by the other members, one having suffered for more than six months. He thought Dr. Opie's method of squeezing the gland a good one in suitable cases.

Dr. C. O'Donovan, Jr., reported

A CASE OF PELVIC ABSCESS, FOLLOWING A FALL DURING PREGNANCY, AND DISCHARGING AT THE UMBILICUS.

On October 29th, 1883, Mrs. F. came to my office and gave the following history of her case:

About the middle of the preceding August, when she was near her time of delivery of her second child, everything being in good condition, and herself in the best of health and spirits, she tripped her foot while going down a flight of steps and pitched headlong about six feet to the floor below, bruising herself considerably by the fall, but retained sufficient consciousness to remount the steps to the room above, when she fell unconscious upon the floor, probably only in a faint. Her frightened family carried her immediately to bed, but she grew worse and worse, becoming feverish and flighty in a few hours, from which condition she fell into a state of complete unconsciousness for five days. A physician in the neighborhood had been called in, who treated her judiciously, expecting miscarriage, but endeavoring by rest and sedatives to make every preparation for the anticipated trouble. This, however, did not occur, but the woman gradually grew quiet, then slowly regained consciousness, and, after the lapse of a full month, she was delivered of a child, exhibiting no marks of injury, although it lived but twelve hours. The puerperal period passed satisfactorily until the third day, when she had a severe chill, followed by a rapid rise of temperature accompanied by a violent pain in the pelvis and lower part of the abdomen. For this her physician treated her locally with large poultices over the abdomen and internal medication, probably anodynes, until the inflammation subsided somewhat, and became more localized, when he ordered turpentine blisters over the sore spots with continuance of the internal treatment. This continued for six weeks, or thereabout, during which time her local trouble seemed to be somewhat better, but her general health was constantly growing worse, so that when I saw her at my office I was shocked at the change I noticed in her. I had known her well, and, although I had heard of her accident, I was totally unprepared for the weak, thin, anæmic woman she now had become from the hearty, vigorous one of three months before. However she managed to get to the office I cannot tell, for so very weak was she that I feared she would be unequal to the task of getting home. She came with her mother and sister, who talked for her, but she sat during the recital of her case, utterly listless, seeming to be the one least interested in her condition and satisfied that nothing could save her from speedy death. She had no appetite, and was unwilling to force herself to eat; milk she loathed, and any simple food sickened her immediately, and most likely was vomited; she craved nothing but ice water, of which she could never get enough. She complained only of an intense, overpowering sense of weakness and fatigue that would allow her to make no exertion whatever, and a heavy, dull pain in the lower part of her abdomen and back. Her pulse was 120, weak, thready and readily compressible. Her respiration was shallow and painful, and with the slightest exertion became hurried. Her expression of face was that of one suffering constantly, lacking animation, careworn and with the fear of death plainly written on her features. She complained also of a constant succession, at more or less regular intervals, of chills, slight sometimes, sometimes severe, but always followed by a fever and profuse sweat. Her bowels were irregular, often constipated, but, at intervals, quite loose.

Beyond this I was unwilling to go, but satisfied myself with hurrying her home, with strict orders to go to bed at once and stay there, giving directions for her nourishment, of milk, chiefly, with plenty of alcoholic stimulants. I saw her, in bed, the next day, and found her, as I expected, very much weakened, by her foolish excursion to my office, but otherwise unchanged. Upon examination an immense pelvic abscess was revealed, consequent, most likely, upon the cellulitis, and evidently pointing at the umbilicus, above which it extended on her left side. The whole pelvis was full of pus, which had pushed the uterus down until the vagina was almost obliterated. I ordered her a poultice to be applied about the umbilicus, and a tonic mixture containing iron. That night the abscess burst at the umbilicus, discharging large quantities of pus, the amount of which could not be ascertained. From that moment she began to improve; she accepted nourishment in small quantities at first, to be gradually increased; her spirits recovered somewhat their usual elasticity; her hectic rapidly diminished; her respiration became fuller and painless. On November 8th her pulse was 108, full and regular, and was rapidly convalescing. I may add that she has never been sick since, and has been delivered of two healthy children at term without difficulty.

This case is interesting for two reasons, chiefly; that it shows the remarkable constitution of the sufferer in whose pelvis and abdomen so large a collection of pus was tolerated for a long time without producing any permanent injury; and that it shows, again, how useless, and perhaps even harmful, is meddling interference with nature when her processes are tending to a satisfactory end; additional safeguards, such as iodoform, washing out with carbolized water, or drainage through the vagina, could not have given a better result than was obtained without them.

